# Generation of dual mouse models of retinal degeneration with slow and rapid progression driven by ectopic RIP3 expression

**DOI:** 10.1371/journal.pone.0356284

**Published:** 2026-08-20

**Authors:** Young Do Song, Jiyeong Kim, Jun-Ho Jang, Youngjin Kim, Hye-Kyoung Oh, Min Jeong Ji, Minji Choi, Minsun Park, Dae-Won Kim

**Affiliations:** 1 Department of Biochemistry, College of Life Science and Biotechnology, Yonsei University, Seoul, Republic of Korea; 2 ICM Company Limited, Seoul, Republic of Korea; Mayo Clinic Minnesota, UNITED STATES OF AMERICA

## Abstract

Dry age-related macular degeneration (AMD) is a leading cause of blindness, characterized by progressive loss of retinal pigment epithelium (RPE) and subsequent photoreceptor degeneration. Current experimental models, including sodium iodate-induced injury, fail to fully recapitulate the chronic, age-related progression of the human disease. Although RIP3-mediated necroptosis has been strongly implicated in RPE cell death, its direct contribution to retinal degeneration in vivo remains unclear. To address this limitation, we generated two RIP3 transgenic mouse lines with distinct patterns of RIP3 overexpression. While RIP3-Tg mice exhibit systemic RIP3 overexpression, RIP3-Tg-RPE mice display additional RPE-specific overexpression beyond the levels observed in RIP3-Tg mice. We then evaluated these transgenic lines, along with wild-type controls, for age-driven retinal degeneration by using optical coherence tomography (OCT), behavior-based visual function assays, and molecular profiling of inflammation and cell death. First, RIP3-Tg mice exhibited gradual retinal thinning, progressive visual decline, and sustained upregulation of pro-inflammatory cytokines (IL-1β, TNF-α, and IL-6) over 6–15 months, recapitulating the slow progression of dry-AMD. Second, RIP3-Tg-RPE mice, which exhibit further RPE-specific increases in RIP3 expression, showed markedly accelerated retinal degeneration, with significant structural and functional deficits evident as early as 2 months of age. These findings indicate that ectopic RIP3 expression in the RPE contributes to inflammatory responses and subsequent retinal degeneration. Collectively, our results highlight RIP3 as a potential contributing factor in the progression of retinal degeneration and introduce biologically relevant transgenic models that capture both slow and accelerated disease progression. These models provide a valuable platform for investigating disease mechanisms and developing therapeutic strategies targeting necroptosis in dry-AMD.

## Introduction

Age-related macular degeneration (AMD) is a leading cause of vision loss worldwide and is characterized by progressive dysfunction and degeneration of the retinal pigment epithelium (RPE) followed by secondary photoreceptor loss [1,2]. Despite its high prevalence, therapeutic options for dry-AMD remain limited, highlighting the need for a better understanding of its underlying disease mechanisms [3–5]. Although oxidative stress and inflammation are well-established contributors to AMD pathogenesis, defining the regulated cell death pathways associated with RPE degeneration and geographic atrophy (GA) remains important for therapeutic development [6].

A variety of experimental models have been developed to study retinal degeneration, including chemical injury models such as sodium iodate-induced and high-intensity light-induced RPE damage models [7]. These models are useful for inducing rapid and reproducible retinal injury, but they do not fully recapitulate the chronic, and progressive nature of human dry-AMD or the complex interplay between cell death and inflammation. Because no single mouse model fully reproduces the anatomical and temporal complexity of human AMD, models that capture selected AMD-relevant features can provide useful mechanistic insight into specific disease-associated pathways.

Programmed cell death pathways have been implicated in RPE degeneration, among which necroptosis has emerged as an important mechanism linking cell death and inflammation. Necroptosis is a regulated form of necrotic cell death mediated by receptor-interacting protein kinases, including RIP3, and is associated with activation of MLKL and the release of pro-inflammatory signals [8–10]. Accumulating evidence suggests that RIP3-MLKL pathway activation contributes to RPE injury under pathological conditions. Increased RIP3 expression has been observed in the maculae of patients with GA as well as in multiple models of retinal injury, suggesting a close association between RIP3 activation and RPE degeneration [3,11]. Consistent with these findings, genetic or pharmacological inhibition of RIP3 has been shown to attenuate RPE loss [12,13]. However, whether sustained increases in RIP3 expression are associated with progressive retinal pathology *in vivo* remains incompletely understood.

To address this gap, we developed complementary RIP3 transgenic mouse models to examine retinal pathology associated with different levels and patterns of ectopic RIP3 expression. The RIP3-Tg line exhibits basal ectopic RIP3 expression and develops slowly progressive retinal degeneration, whereas the RIP3-Tg-RPE line carries this basal expression together with additional RPE-enriched RIP3 expression driven by Best1-Cre and displays accelerated retinal degeneration. Thus, these models were designed to investigate gradually progressive and accelerated forms of RIP3-associated retinal pathology, rather than to serve as complete phenotypic replicas of human AMD.

In this study, we evaluated structural, histological, behavioral, and molecular changes associated with increased RIP3 expression using longitudinal imaging, tissue analysis, behavioral assessments, and inflammatory and necroptosis-associated marker profiling. Through these approaches, we aimed to determine whether increased RIP3 expression burden is associated with retinal degeneration, inflammatory activation, and RIP3-MLKL pathway activation, and to establish biologically relevant models for studying selected AMD-like retinal pathology.

## Methods

### Mice and human RPE tissue

**Fig 1:**
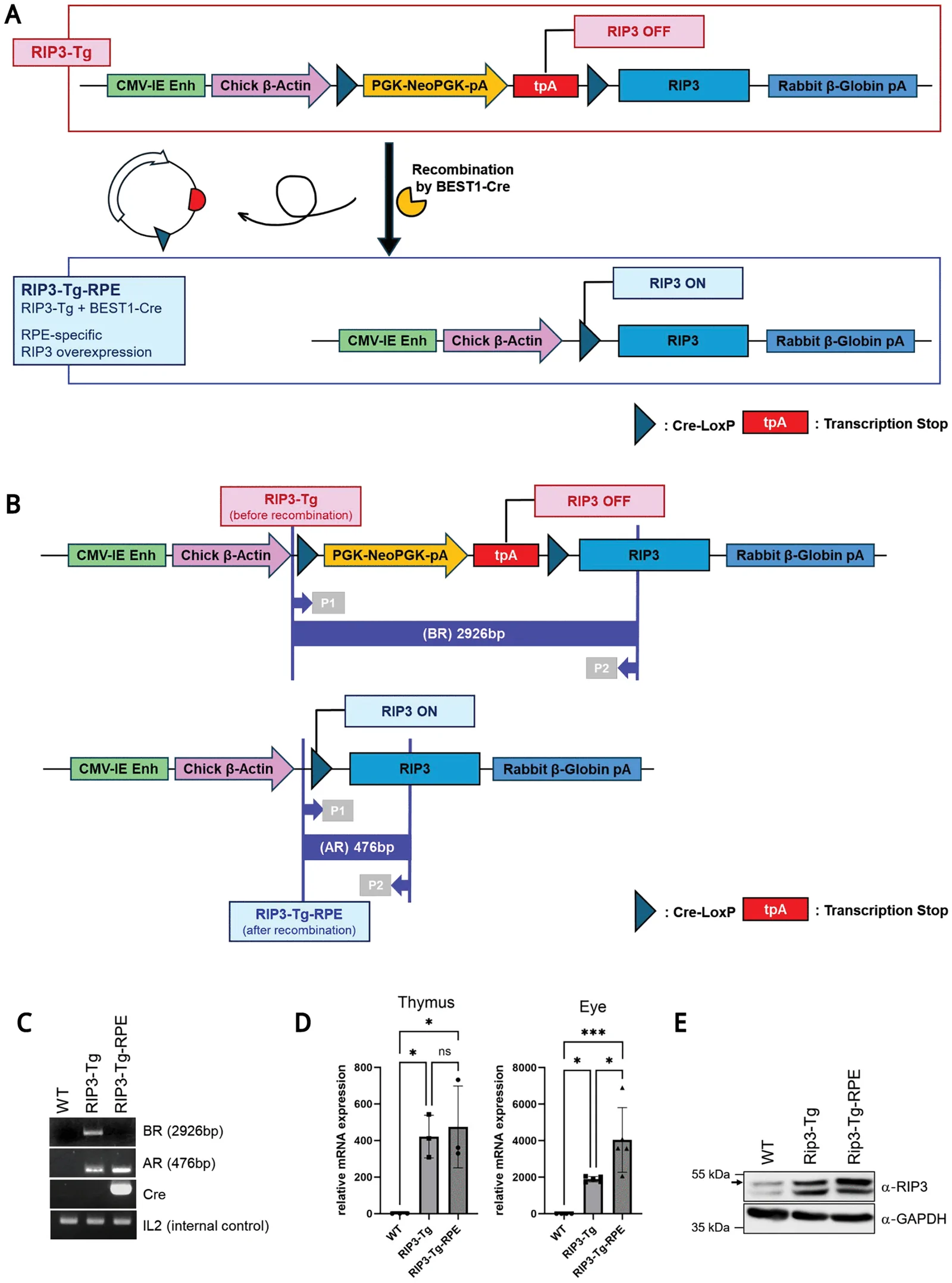
Generation of RIP3 overexpression mice. **(A)** Schematic diagram of Cre-inducible RIP3 transgenic mouse model. The pCB-RIP3 transgenic construct was generated by subcloning RIP3 coding sequence into the multiple cloning site of the pCB vector. The pCB vector was assembled by inserting a loxP-flanked transcriptional STOP cassette (tpA; triple SV40-polyadenylation sequence) from the pBigT vector downstream of the robust CAG promotor in the pCAGGS vector. In the resulting conditional RIP3-Tg mice, baseline transgenic RIP3 expression is inhibited by the tpA cassette in the absence of Cre recombinase. To achieve RPE-specific overexpression, RIP3-Tg mice were crossed with Best1-Cre mice. Cre-mediated recombination excises the loxP-flanked tpA cassette (indicated by the spiral arrow), allowing the CAG promoter to drive the expression of the RIP3 transgene in the RIP3-Tg-RPE mice. **(B)** The schematic describes the expression construct and the specific binding sites for primers P1 and P2, which were utilized to confirm the presence or absence of LSL(loxP-STOP-loxP) cassette excision. Amplification of the intact (before recombination, BR) allele yields a 2926-bp PCR product, whereas the Cre-mediated recombined allele (after recombination, AR) produces a 476-bp fragment. lines. **(C)** PCR genotyping analysis of offsprings derived from crosses between RIP3-Tg and Best1-Cre lines. **(D)** RT-qPCR analysis of RIP3 mRNA expression in 2-month-old thymus and eyes from WT, RIP3-Tg, and RIP3-Tg-RPE mice. Data shown were evaluated by one-way ANOVA test. Asterisks represent statistical analyses compared to the WT controls *P < 0.05, **P < 0.01, ***P < 0.001. RT-qPCR data listed in S1 Table. (Thymus: n = 3 samples/group, Eyes: n = 4–5 samples/group) **(E)** Western blot (WB) analysis for RIP3 expression in eyes of 2-month-old WT and RIP3-Tg mice. (n = 3 per group). Arrow indicates the band representing RIP3.

C57BL6 mice were used to generate RIP3-Tg and RIP3-Tg-RPE mice and the overall scheme and genotyping strategy are shown in Fig 1A. Mouse genotyping was performed as previously described, and the primer sequences are listed in Table 1. All animal care procedures adhered to the guidelines set by the ARVO Statement for the Use of Animals in Ophthalmic and Vision Research and were approved by the Institutional Animal Care and Use Committee of Yonsei Laboratory Animal Research Center (permit no. IACUC-A-202011-1175-02). Human donor eyes (n = 4) were obtained from the Center for Vision and Eye Banking Research Eversight (Chicago, IL, USA, and Ann Arbor, MI, USA) within 12 hrs after death. Detailed information regarding human donors are listed in Table 2.

**Table 1:** Primer sequences used for genotyping analyses.

Gene	Primers	
BEST1 (cre)	Forward	5’ CCA TCT GCC ACC AGC CAG 3’
	Reverse	5’ TCG CCA TCT TCC AGC AGG 3’
IL-2 (internal control)	Forward	5’ CTAGGCCACAGAATTGAAAGATCT 3’
	Reverse	5’ GTAGGTGGAAATTCTAGCATCATCC 3’
P1/P2 (recombination assessment)	Forward	5’ AGT GGA TCC GGA ACC CTT AAT 3’
	Reverse	5’ GAG GGT CCA AGC TGT GTA GG 3’

Abbreviation: *BEST1: Bestrophin 1 promoter-inducible Cre, IL-2: Interleukin 2.*

**Table 2:** Detailed information regarding human retinal donors.

Subject	Donor age	Donor Gender	Ophthalmic record	Tissue Processing	Assessment
A	39	Male	None	RPE-Choroidal	WB
B	27	Male	None	RPE-Choroidal	WB
C	80	Male	None	RPE-Choroidal	WB
D	80	Male	None	RPE-Choroidal	WB

### RNA isolation and quantitative real-time PCR

Total RNA was isolated using GE illustra RNAspin mini kit (GE Healthcare, Chicago, USA) according to the manufacturer’s instructions. The purity and concentration of the RNA samples were assessed using a NanoDrop spectrophotometer (Thermo Fisher Scientific, Waltham, USA), with A_260/280_ ratio ranging from 1.8 to 2.0 considered acceptable. cDNA was synthesized from extracted RNA using TOPscript^TM^cDNA synthesis kit (Enzynomics, Daejeon, Korea). Quantitative PCR was performed using the StepOnePlus Real-Time PCR System using SYBR Green PCR Master Mix (Thermo Fisher Scientific, Massachusetts, USA). The primers sequences are listed in Table 3.

**Table 3:** Primer sequences used in RT-qPCR analyses.

Gene	Primers	
IL-1β	Forward	CCA CCA ACA AGT GAT ATT CTC CAT G
	Reverse	GTG CGG TCT TTC ATT ACA CAG
TNF-α	Forward	CAC AAG ATG CTG GGA CAG TGA
	Reverse	TCC TTG ATG GTG GTG CAT GA
IL-6	Forward	GCC TTC TTG GGA CTG ATG CT
	Reverse	GAC AGG TCT GTT GGG AGT GG
RIP3	Forward	TGT CAA GTT ATG GCC TAC TGG TGC G
	Reverse	AAC CAT AGC CTT CAC CTC CCA GGA T
GAPDH	Forward	ATA CGG CTA CAG CAA CAG GG
	Reverse	GCC TCT CTT GCT CAG TGT CC

Abbreviation: *IL-1β: Interleukin 1 Beta, TNF-α: Tumor necrosis factor alpha, IL-6: Interleukin 6, RIP3: Receptor-interacting serine/threonine-protein kinase 3, GAPDH: Glyceraldehyde 3-phosphate dehydrogenase.*

### Histology and immunohistochemistry (IHC)

Mice were euthanized and whole eyes were fixed in 4% (w/v) paraformaldehyde (PFA) in PBS for 7 hrs at RT, followed by 2-hr fixation with Davidson’s solution. The fixed eyes were then processed and embedded in paraffin before sectioning at 4 µm and mounted on silane-coated slides (Muto-Glass, Tokyo, Japan). For deparaffinization, sections were treated sequentially with xylene, a xylene/ethanol mixture, and serial ethanol solutions. Antigen retrieval was performed by incubating sections in EDTA (pH 9.0) and 0.05% Tween 20 at 25 °C for 20 min, followed by washing in PBS. Endogenous peroxidase activity was blocked by incubating sections in 3% H_2_O_2_ at 60°C for 30 min. Then, blocked with blocking buffer containing 5% (v/v) goat (or bovine) serum, 0.05% sodium azide, and 0.1% TiritonX-100 in PBS for 1h at 25 °C. Sections were then incubated overnight at 4 °C with the indicated primary antibodies diluted in antibody dilution solution buffer containing 1% (v/v) goat (or bovine) serum, 0.05% sodium azide, and 0.02% Tween 20. After washing, sections were incubated for 1h at RT with either Alexa594-conjugated anti-rabbit IgG or Alexa488-conjugated anti-goat IgG secondary antibodies (Cell Signaling, Denver, USA), diluted in the same antibody buffer. Slides were mounted using a homemade anti-fading solution consisting of 25mM Tris(pH8.7), 10% polyvinyl alcohol, 5% glycerol, and 2.5% DAMCO (Sigma, Massachusetts, USA). To confirm the morphology, serial sections were stained with Gill’s hematoxylin and eosin (H&E). The H&E images were taken from fluorescent microscope (Zeiss, Oberkochen, Germany). Fluorescent imaging data were acquired using a Leica Stellaris 5 confocal microscope equipped with LAS X software. Information regarding the antibodies used in this study is provided in Table 4.

**Table 4:** Antibody used in western blot and immunohistochemistry analyses.

Target antigen	Vendor or Source	Catalog #	Working
			concentration
RIP3	Enzo Life Sciences	ADI-905-242-100	WB 1:1000, IF 1:300
GAPDH	Abfrontier	LF-PA0018	WB 1:5000
RPE65	BD Biosciences	552850	IF 1:100
p-MLKL	Cell Signaling	37333	IF 1:200
RIP3	Cell Signaling	13526	WB 1:1000
p-RIP3	Abcam	Ab222320	WB 1:1000
p-MLKL	Abcam	Ab187091	WB 1:1000
Phalloidin	Abcam	Ab176753	IF 1:5000

Abbreviations: *WB: Western blot, IF: Immunofluorescence, RIP3: Receptor-interacting serine/threonine-protein kinase 3, GAPDH: Glyceraldehyde 3-phosphate dehydrogenase, RPE65: retinal pigment epithelium-specific 65, p-MLKL: phosphorylated mixed lineage kinase domain-like protein, p-RIP3: phosphorylated receptor-interacting serine/threonine-protein kinase 3.*

### RPE flatmount

To evaluate the morphology and RIP3 expression in the RPE monolayer, eyes were enucleated and fixed in 4% (w/v) PFA in PBS for 30 min at RT. The cornea, lens, and retina were carefully removed to isolate the RPE choroid complex. The RPE choroid complex was then cut to eight radial incisions to create a flatmount and incubated in blocking buffer (5% (v/v) goat serum and 0.3% Triton-X-100 in PBS) for 1h at RT. Flatmounts were incubated overnight at 4 °C with primary antibodies. After washing with PBS, samples were incubated for 1h at RT with Alexa Fluor conjugated secondary antibodies. To visualize the RPE cell boundaries, flatmounts were co stained with phalloidin (Invitrogen, Massachusetts, USA). Finally, the samples were mounted with the homemade anti-fading solution described above and examined using a Leica Stellaris 5 confocal microscope.

### Protein extraction and western blot analysis

Isolation of RPE cell protein from mouse eyes was conducted following a previously established method [14]. Equal amounts of protein were resolved by sodium dodecyl sulfate-polyacrylamide gel electrophoresis (SDS-PAGE) and transferred onto a nitrocellulose membrane (Millipore, California, USA). The membrane was blocked with 5% skimmed milk diluted in PBS with 0.05% Tween 20 (PBST) at room temperature (RT) for 1h, followed by incubation with indicated primary antibodies at 4°C overnight. After washing, membranes were incubated with horseradish peroxidase (HRP)-conjugated secondary antibodies at RT for 30 min and developed using EZ-Western Lumio enhanced chemiluminescence (ECL) and Lumi Femto kit (DoGenBio, Seoul, Korea). ECL signals were detected using an Amersham Imager 680 (GE Healthcare). Intensities were normalized to the housekeeping gene such as GAPDH. Information regarding the antibodies used in this study is provided in Table 4.

### Fundus photography and optical coherence tomography

Fundus images and optical coherence tomography(OCT) scans were taken using the MICRON IV (Phoenix-Micron, Oregon, USA). Retinal thickness was measured using InSight-Animal OCT Segmentation Software (Phoenix-Micron).

### TUNEL assay

Cell death was examined in eye sections using an *in-situ* cell death detection kit (Roche Applied Science, Indianapolis, USA) according to the manufacturer’s protocol. To preferentially detect necroptotic cell death, we omitted the standard permeabilization step (such as Triton-X-100 treatment) typically used in this protocol. By bypassing this step, the terminal deoxynucleotidyl transferase enzyme only accesses DNA fragments in cells where the plasma membrane integrity has been lost, which is a characteristic feature of necroptotic cell death. Briefly, the sections were incubated with 1% sodium citrate for 15 min, then rinsed with PBS, incubated with TUNEL reagents for 1h at 37°C, and washed in PBS for 5 min. Slides were mounted using a homemade anti-fading solution. TUNEL imaging data were acquired and processed using a Leica Stellaris 5 confocal microscope equipped with LAS X software.

### Open Field Mirror Test (OFMT)

The open field mirror test was performed following a previously described protocol [15]. The experimental arena was conceptually divided into three principal regions—the mirrored zone, the reversed zone, and the null zone—each comprising specific subzones measuring 16.67 × 16.67 cm (S2 Fig). A reflective mirror (15 × 15 cm) was mounted centrally on the wall corresponding to zone 2. Directly opposite, in zone 8, a non-reflective, reversed mirror of identical dimensions and texture was installed to serve as a control object. The intermediate space (zones 4, 5, and 6) contained no objects and was designated as the null zone.

Testing order was randomized across the experimental mouse groups. For each trial, the individual mouse was placed in the center of the arena, and its behavior was continuously recorded for 10 min using a digital video camera. The recorded footage was subsequently analyzed to quantify the following behavioral parameters: (i) total distance traveled, (ii) duration of time spent in each specific zone, and (iii) the frequency of zone entries.

### Statistical analysis

All statistical analyses were performed using GraphPad Prism 10 (GraphPad Software, California, USA). Data are expressed as the mean ± standard error of the mean (SEM). Each experiment was conducted at least 3 times, and representative images or pooled data are shown in the figures. Statistical comparisons were evaluated using two-tailed unpaired t-tests, multiple unpaired t-tests, one-way ANOVA, or two-way ANOVA, assuming normality and homogeneity of variance for all data. P < 0.05 was considered a statistically significant difference.

## Results

### Generation of RIP3-overexpressing transgenic mice

To generate an RPE-specific conditional transgenic model for RIP3 overexpression, RIP3-Tg mice harboring a Cre-inducible loxP-STOP-loxP *Rip3* cassette were crossed with Best1-Cre mice expressing Cre recombinase under the control of the RPE-specific *Best1* promoter. This breeding strategy generated four experimental groups: wild-type (WT), RIP3-Tg, Best1-Cre, and the double-transgenic RIP3-Tg-RPE mice.

During the initial characterization of the RIP3-Tg line, we observed exogenous RIP3 transgene expression even in the absence of Cre-recombinase activity, suggesting basal transgene leakage from this construct. To verify the transgene recombination status, we designed a pair of primers flanking the two loxP sites and performed genomic PCR analysis in RIP3-Tg and WT (Fig 1A and 1B). These PCR analyses revealed that RIP3-Tg exhibit both the pre-recombination (2926 bp) and post-recombination alleles (476 bp), whereas no corresponding bands were detected in WT (Fig 1C). The presence of both alleles in RIP3-Tg indicates Cre-independent loxP recombination, resulting in genetic mosaicism.

To confirm transgene expression in the generated mouse lines, *RIP3* mRNA levels were quantified by RT-qPCR in both the eye, the target tissue, and the thymus, a well-established RIP3-expressing tissue. Consistent with the genotyping results, both RIP3-Tg and RIP3-Tg-RPE mice exhibited elevated *RIP3* expression in the thymus compared to WT controls (Fig 1D, left, S1 Table). Similar expression levels between the two transgenic lines were observed as expected (421-fold vs 474-fold), since the Best1 promoter does not drive Cre expression in the thymus. In the eye, RIP3 expression was increased in both transgenic lines relative to WT mice, but, notably, RIP3-Tg-RPE mice displayed markedly higher (1847-fold vs 4534-fold) RIP3 expression than RIP3-Tg mice (Fig 1D, right, S1 Table). Consistent with these transcriptional changes, western blot analysis confirmed a significant increase in RIP3 protein levels in both RIP3-Tg and RIP3-Tg-RPE mice compared to WT controls. Mirroring the mRNA profile, RIP3-Tg-RPE mice exhibited higher RIP3 protein accumulation than the RIP3-Tg mice (Fig 1E). These findings confirm successful RIP3 transgene expression in both models at both the transcriptional and translational levels, with enhanced ocular expression in the RPE-targeted line (RIP3-Tg-RPE).

### Progressive retinal degeneration in RIP3-Tg mice

Our initial characterization of the RIP3-Tg line revealed an unexpected yet robust model for studying the effects of RIP3 overexpression independent of Best1-Cre–mediated recombination. Although RIP3 overexpression has been reported to cause minimal phenotypic changes *in vivo*, its impact on retinal structure and function has not been systematically examined. Therefore, prior to evaluating the RPE-specific model, we first investigated the RIP3-Tg line to determine whether spontaneous Cre-independent RIP3 overexpression influences retinal degeneration.

**Fig 2:**
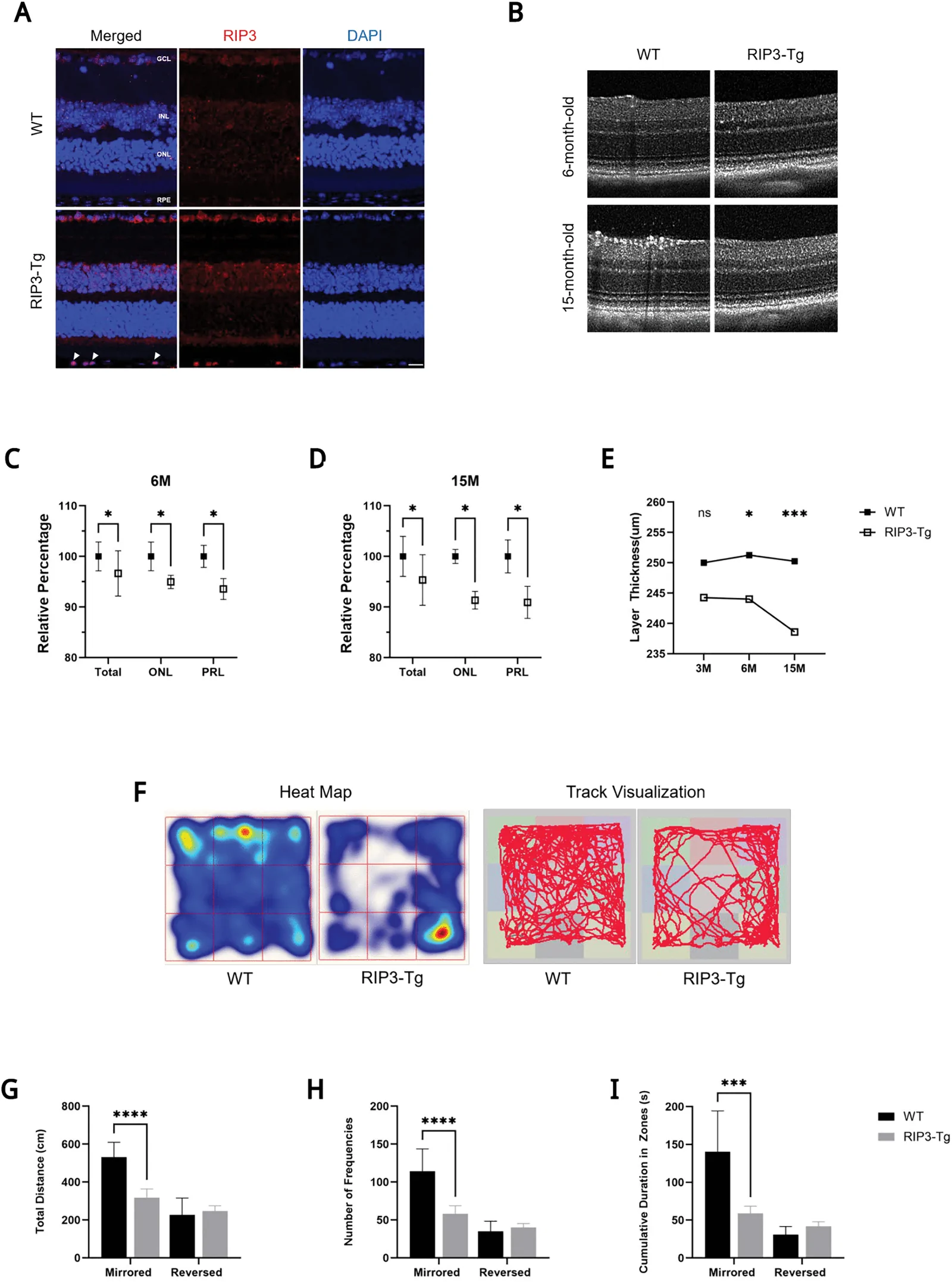
Progressive retinal degeneration in RIP3-Tg mice. **(A)** Immunofluorescence(IF) analysis for RIP3 (red) and DAPI (blue) staining in 2-month-old mice retina. An increase in RIP3-staining was detected in RIP3-Tg retina. Arrows indicate strong RIP3 signal. **B-E:** Fundus and OCT images of WT and RIP3-Tg and quantitative comparison of layer thickness. **(B)** Fundus and OCT images of 6-month-old and 15-month-old eyes of WT and RIP3-Tg mice. C-E Quantitative comparison of relative Total, ONL, and PRL thickness from OCT images from **(C)** 6-month-old WT (n = 8/group) and RIP3-Tg mice (n = 12/group). **(D)** 15-month-old WT (n = 8/group) and RIP3-Tg mice (n = 12/group). Thickness values are expressed as percentages relative to the WT controls (set to 100%). Data shown were evaluated by multiple unpaired t-test. Asterisks represent statistical analyses compared to the WT controls. *P < 0.05. Total, ONL, and PRL thickness statistical data listed in Table 5. Layer thickness measurements listed in S2 Table. **(E)** Quantitative comparison of retinal layer thickness from OCT images of 3, 6, and 15-month-old WT (n = 8/group) and RIP3-Tg mice (n = 12/group). Data shown were evaluated by 2-way ANOVA test. Asterisks represent statistical analyses compared to the WT controls. *P < 0.05, **P < 0.01, ***P < 0.001. Layer thickness statistical data listed in Table 5. Layer thickness measurements listed in S2 Table. **(F-I)** Open field mirror-test of 15-month-old WT and RIP3-Tg mice. (WT n = 3, RIP3-Tg n = 6). Open-field mirror-test info is explained in S1 Fig. **(F)** Representative heatmap and track visualization from the open-field mirror test. **(G)** Total distance traveled in mirrored or reversed zones. **(H)** Number of frequencies entered mirrored or reversed zones. **(I)** Cumulative duration stayed in mirrored or reversed zones. Data shown were evaluated by 2-way ANOVA test. Asterisks represent statistical analyses compared to WT controls. *P < 0.05, **P < 0.01, ***P < 0.001, ****P < 0.0001. Data measurements listed in S3 Table. ONL: outer nuclear layer; PRL: photoreceptor layer. Scale bars: A: 10μm, C: 50μm.

To validate RIP3 overexpression at the protein level in the retina, we performed immunofluorescence (IF) staining analysis using eyes collected from 2-month-old mice. IF staining revealed elevated RIP3 signals throughout the retinal layers in RIP3-Tg mice (Fig 2A). Next, we evaluated the long-term impact of sustained ectopic RIP3 expression on retinal structural integrity using optical coherence tomography (OCT). Representative OCT images revealed gradual retinal thinning in RIP3-Tg mice over time (Fig 2C), which was confirmed by quantitative analysis (S1 Fig). At 6 months of age, RIP3-Tg mice exhibited significant reductions in total retinal thickness (251 µm vs 244 µm), as well as outer nuclear layer (ONL; 71 µm vs 67 µm) and photoreceptor layer (PRL; 53 µm vs 50 µm) (Fig 2C, Table 5, S2 Table). These structural defects became more pronounced by 15 months of age, with total retinal thickness reduced to 236 µm (compared to 250 µm in WT), ONL thickness to 62 µm (compared to 68 µm), and PRL thickness to 46 µm (compared to 51 µm), indicating a progressive age-dependent retinal degeneration (Fig 2D, Table 5, S2 Table). Longitudinal analysis across 3, 6 and 15 months clearly demonstrated a progressive decline in retinal thickness over time (Fig 2E, Table 5, S2 Table).

**Table 5:** Data table for retina thickness comparison between 3, 6, and 15 months RIP3-Tg compared to WT.

Explanatory variable		P-value	q-value	Degrees of freedom
WT 6M vs RIP3-Tg 6M	Total	0.01184461	0.00398769	54
	ONL	0.00026356	0.0001331	54
	PRL	0.00000667	0.00000674	54
Explanatory variable		P-value	q-value	Degrees of freedom
WT 15M vs RIP3-Tg 15M	Total	0.00378351	0.00382134	54
	ONL	0.00000067	0.00000101	54
	PRL	0.00000025	0.00000077	54
Explanatory variable		P-value	Degrees of freedom	
WT 3M vs RIP3-Tg 3M		0.0937	54	
WT 6M vs RIP3-Tg 6M		0.0228	54	
WT 15M vs RIP3-Tg 15M		0.0001	54	

Finally, to determine whether this progressive retinal thinning leads to visual functional deficits, we performed an open-field mirror test (OFMT) (S2 Fig). As previously described [15], this behavioral paradigm leverages the innate sociability of mice; animals with normal visual acuity preferentially spend more time in mirrored zones, whereas visually impaired mice lack this preference, displaying random exploratory behavior. To assess functional impairment, visual behavior was evaluated in 15-month-old mice. Heatmap and trajectory analyses demonstrated that WT mice predominantly remained within mirrored zones, whereas RIP3-Tg mice lacked this preference and frequently explored non-mirrored zones (Fig 2F). Quantitative analysis further revealed that RIP3-Tg mice exhibited reduced total distance traveled, shorter duration, and fewer entries within mirrored zones compared to WT controls (Fig 2G–I, S3 Table), providing a potential alteration in visual behavior, which serves as an indirect indicator of visual impairment. To address whether these behavioral alterations were influenced by generalized stress or altered emotional states, we quantified the number of fecal pellets, a proxy for anxiety like behavior in rodents (S3 Fig, S4 Table). We observed increased fecal pellets counts in RIP3-Tg mice compared with WT littermates, suggesting that the reduced exploratory activity and shorter total distance traveled may, in part, reflect increased anxiety like behavior in the novel open field environment. However, when integrated with the significant retinal structural degeneration and thinning observed by OCT, these behavioral deficits collectively indicate a progressive decline in visual function. Taken together, these findings demonstrate that basal RIP3 overexpression in RIP3-Tg mice strongly associates with and induces progressive retinal structural degeneration accompanied by visual function decline.

### Histological changes in RIP3-Tg mice

**Fig 3:**
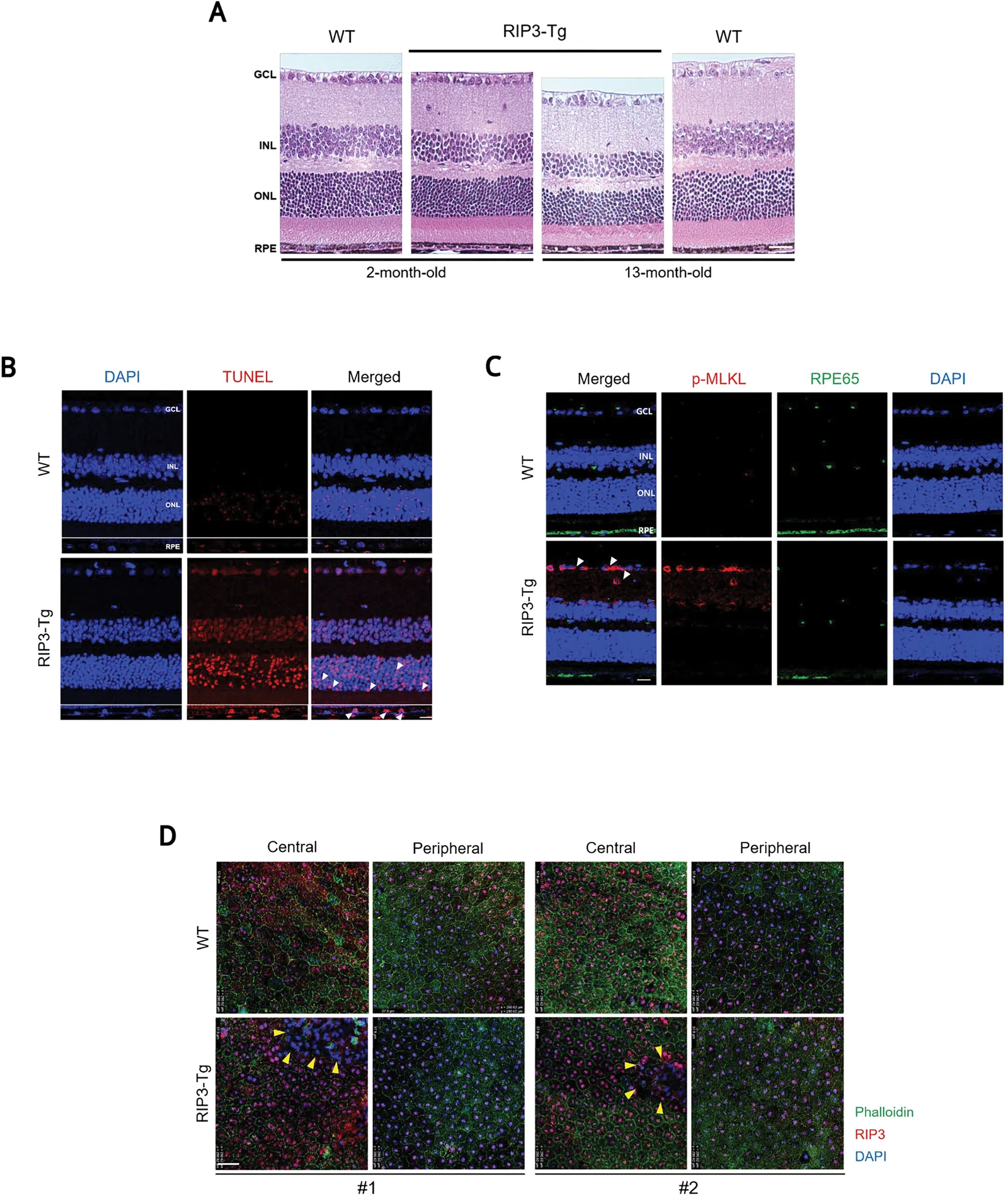
Histological changes in RIP3-Tg mice. **(A)** H&E histology data shows progressive retinal thinning and RPE loss in RIP3-Tg mice compared to WT mice at 13 months (n = 3 for each group and timepoint). H&E: hematoxylin and eosin; RPE: retinal pigment epithelium. **(B)** TUNEL (red) and DAPI (blue) staining in 13-month-old WT and RIP3-Tg retina. Staining is performed without permeabilization. (n = 3/group). Arrows indicate strong TUNEL signal. **(C)** Immunofluorescence staining of p-MLKL(red), RPE65(green), and DAPI(blue) in 13-month-old WT and RIP3-Tg retina. Arrows indicate strong p-MLKL signal. **(D)** Flatmount immunofluorescence staining of phalloidin(green), RIP3(red), and DAPI(blue) in 3-month-old WT and RIP3-Tg mice. (n = 3/group). Arrows indicate localized disruption of the RPE cell layer. Scale bars: A, D: 20 μm, B, C: 10μm.

To validate the longitudinal retinal thinning observed by OCT, we performed histological analysis on retinal sections from 2- and 13-month-old WT and RIP3-Tg mice using hematoxylin and eosin (H&E) staining. At 13 months of age, RIP3-Tg mice exhibited marked disorganization of retinal layers and significant thinning of the ONL and PRL compared with WT controls, confirming progressive structural degeneration (Fig 3A). Given the interplay between retinal degeneration and programmed cell death, we evaluated retinal cell death at 13 months of age using a modified TUNEL assay [16]. To specifically identify necroptotic cells, we performed the assay without cell permeabilization. This approach restricts DNA labeling to cells with compromised plasma membranes, a characteristic feature of necroptosis, thereby minimizing the detection of apoptotic cells with intact membranes. TUNEL positive signals were detected across multiple retinal layers, including the GCL, INL, ONL, and RPE, indicating widespread necroptotic cell death throughout the retina (Fig 3B). To further confirm whether this widespread cell death was mediated by necroptotic pathway, we performed immunofluorescence co-staining for phosphorylated MLKL (p-MLKL), a well-stablished sign of necroptosis, and RPE65, a marker for RPE cells (Fig 3C). In 13-month-old RIP3-Tg mice, prominent punctate staining of p-MLKL was detected in GCL and INL. In contrast, while p-MLKL signals were less evident in the RPE layer at this advanced stage, RIP3-Tg mice exhibited a patchy and irregular loss of RPE65 staining in these same regions.

To determine whether these retinal abnormalities were accompanied by RPE stress, we examined RPE morphology and RIP3 expression in 3-month-old mice. RPE flatmounts stained with phalloidin and RIP3 revealed localized disruption of the RPE cell layer in RIP3-Tg mice (Fig 3D). In the central retina, RIP3-Tg mice showed fragmented cell boundaries and evidence of cell loss, whereas the peripheral retina displayed stretched and elongated RPE cell morphology (Fig 3D). These morphological alterations were associated with ectopic RIP3 expression within the RPE layer. It is important to note that these observations reflect regionally heterogenous RPE disruptions rather than uniform cell loss. Furthermore, to evaluate functional stress in the RPE preceding overt cell loss, western blot analysis of 5-month-old mice confirmed a marked downregulation of RPE65 protein alongside robust RIP3 overexpression in RIP3-Tg mice (S4 Fig). Collectively, these findings demonstrate that RIP3 overexpression is associated with both structural retinal degeneration and RPE-specific functional decline.

### Accelerated retinal degeneration in RIP3-Tg-RPE mice

Based on the progressive phenotype observed in the RIP3-Tg mice, we next investigated RIP3-Tg-RPE model whether enhanced RIP3 expression in the RPE further accelerates retinal degeneration. To this end, we analyzed double-transgenic RIP3-Tg-RPE mice. As demonstrated above, introduction of Best1-Cre markedly increased *RIP3* expression in the eye.

**Fig 4:**
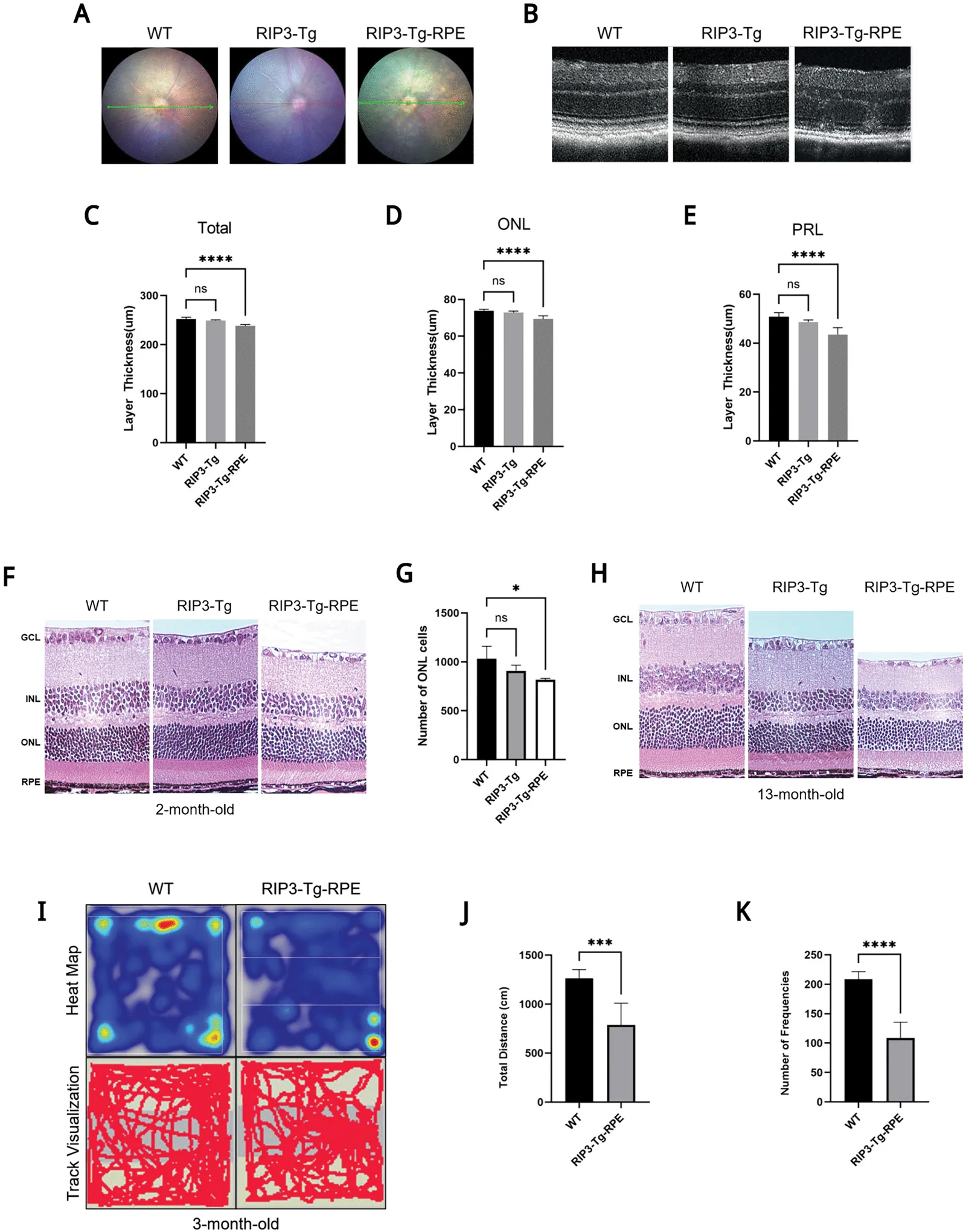
Accelerated retinal degeneration in RIP3-Tg-RPE mice. **(A-B)** Fundus **(A)** and OCT **(B)** images of 2-month-old WT, RIP3-Tg and RIP3-Tg-RPE. (**C-E)** Quantitative comparison of layer thickness from OCT images of 2-month-old WT, RIP3-Tg and RIP3-Tg-RPE mice (n = 5–11/each group). **(C)** Total retina layer thickness. **(D)** ONL thickness. **(E)** PRL thickness. Data shown were evaluated by one-way ANOVA test. Asterisks represent statistical analyses compared to the WT controls *P < 0.05, **P < 0.01, ***P < 0.001, ****P < 0.0001. Statistical data of Total, ONL, PRL listed in Table 6, Table 7, Table 8, respectively. Layer thickness measurements of Total, ONL, PRL listed in S5 Table, S6 Table, S7 Table, respectively. **(F)** H&E histology evaluation at 2 months of age revealed a progressive decline in ONL cellularity in RIP3-Tg-RPE mice (n = 5/each group). **(G)** Quantitative comparison of number of ONL cells from 2-month-old H&E histology images. Data shown were evaluated by one-way ANOVA test. Asterisks represent statistical analyses compared to the WT controls *P < 0.05. Statistical data listed in Table 9. Number of ONL cell counts listed in S8 Table. **(H)** H&E histology evaluation at 13 months of age revealed accelerated decline in ONL cellularity in both RIP3-Tg and RIP3-Tg-RPE mice (n = 5/each group) **(I)** Open-field mirror test of 3-month-old WT, RIP3-Tg and RIP3-Tg-RPE mice (n = 6/each group). Representative heat map and track visualization from the open-field mirror test. **(J)** Total distance traveled in mirrored zone **(K)** Cumulative duration stayed in mirrored zone. Data shown were evaluated by two-tailed unpaired t-test. Asterisks represent statistical analyses compared to the WT controls. *P < 0.05, **P < 0.01, ***P < 0.001, ****P < 0.0001. Data measurements listed in S9 Table. ONL: outer nuclear layer.

To determine the *in vivo* morphological consequences of enhanced RIP3 expression, fundus and OCT imaging were performed. Representative fundus images (Fig 4A) and corresponding OCT scans (Fig 4B) revealed clear structural differences between the two models at early stages. Quantitative analysis of total retina (Fig 4C, Table 6, S5 Table), ONL (Fig 4D, Table 7, S6 Table), and PRL (Fig 4E, Table 8, S7 Table) thickness demonstrated markedly accelerated degeneration in RIP3-Tg-RPE mice. Notably, while RIP3-Tg mice exhibited no significant structural changes at 2 months of age, RIP3-Tg-RPE mice displayed pronounced retinal thinning as early as this time point.

**Table 6:** Data table for Total thickness comparisons between 2-month-old RIP3-Tg, or RIP3-Tg-RPE compared to WT.

Explanatory variable	P-value	q-value	Degrees of freedom
WT 2M vs RIP3-Tg 2M	0.2843	1.409	18
WT 2M vs RIP3-Tg-RPE 2M	<0.0001	8.802	18

**Table 7:** Data table for ONL thickness comparisons between 2-month-old RIP3-Tg, or RIP3-Tg-RPE compared to WT.

Explanatory variable	P-value	q-value	Degrees of freedom
WT 2M vs RIP3-Tg 2M	0.4304	1.120	18
WT 2M vs RIP3-Tg-RPE 2M	<0.0001	5.855	18

**Table 8:** Data table for PRL thickness comparisons between 2-month-old RIP3-Tg, or RIP3-Tg-RPE compared to WT.

Explanatory variable	P-value	q-value	Degrees of freedom
WT 2M vs RIP3-Tg 2M	0.2497	1.492	18
WT 2M vs RIP3-Tg-RPE 2M	<0.0001	5.883	18

Histological analysis using H&E staining further supported these findings (Fig 4F). At 2 months of age, RIP3-Tg-RPE mice exhibited marked ONL thinning and cellular loss compared to WT mice, which was confirmed by quantitative analysis of ONL nuclei (Fig 4G, Table 9, S8 Table). In contrast, RIP3-Tg mice remained comparable to WT controls at this early stage. However, by 13 months of age, both transgenic lines exhibited severe ONL degeneration relative to WT mice, consistent with advanced disease progression (Fig 4H). These findings indicate that while the RIP3-Tg model develops retinal degeneration progressively over time, the RIP3-Tg-RPE model, which exhibits additional RPE-specific RIP3 overexpression, markedly accelerates disease onset, with clear structural abnormalities evident as early as 2 months of age.

**Table 9:** Data table for number of ONL cells comparisons between 2, and 13 months RIP3-Tg, or RIP3-Tg-RPE compared to WT.

Explanatory variable	P-value	q-value	Degrees of freedom
WT 2M vs RIP3-Tg 2M	0.1275	3.089	9
WT 2M vs RIP3-Tg-RPE 2M	0.0115	5.299	9

To evaluate the functional consequences of this accelerated degeneration, we performed the open-field mirror test in 3-month-old mice. Heatmap and trajectory analyses demonstrated that WT preferentially remained in the mirrored zones, whereas RIP3-Tg-RPE mice lacked this preference and showed random exploratory behavior (Fig 4I). Quantitative analysis further revealed reduced total distance and fewer entries into mirrored zones in RIP3-Tg-RPE mice compared to WT controls, which collectively provide indirect functional evidence of altered behavioral performance (Fig 4J and K, S9 Table). Fecal pellets counting was also performed to assess exploratory patterns and potential anxiety related factors (S5 Fig, S10 Table). Consistent with the findings in the RIP3-Tg model, we observed increased fecal pellets counts in RIP3-Tg-RPE mice, suggesting that these behavioral observations may be modulated by the heightened emotional distress of the animals in the novel testing arena.

Collectively, these data suggest that increased RIP3 expression in the RPE markedly accelerates retinal degeneration, supporting a functional link between RIP3 activity and disease progression. While the RIP3-Tg model reflects slow, age dependent degeneration, the RIP3-Tg-RPE model recapitulates a rapidly progressive form of retinal degeneration resembling key features of dry-AMD.

### Inflammatory and RIP3-MLKL pathway signatures in RIP3-associated retinal degeneration

**Fig 5:**
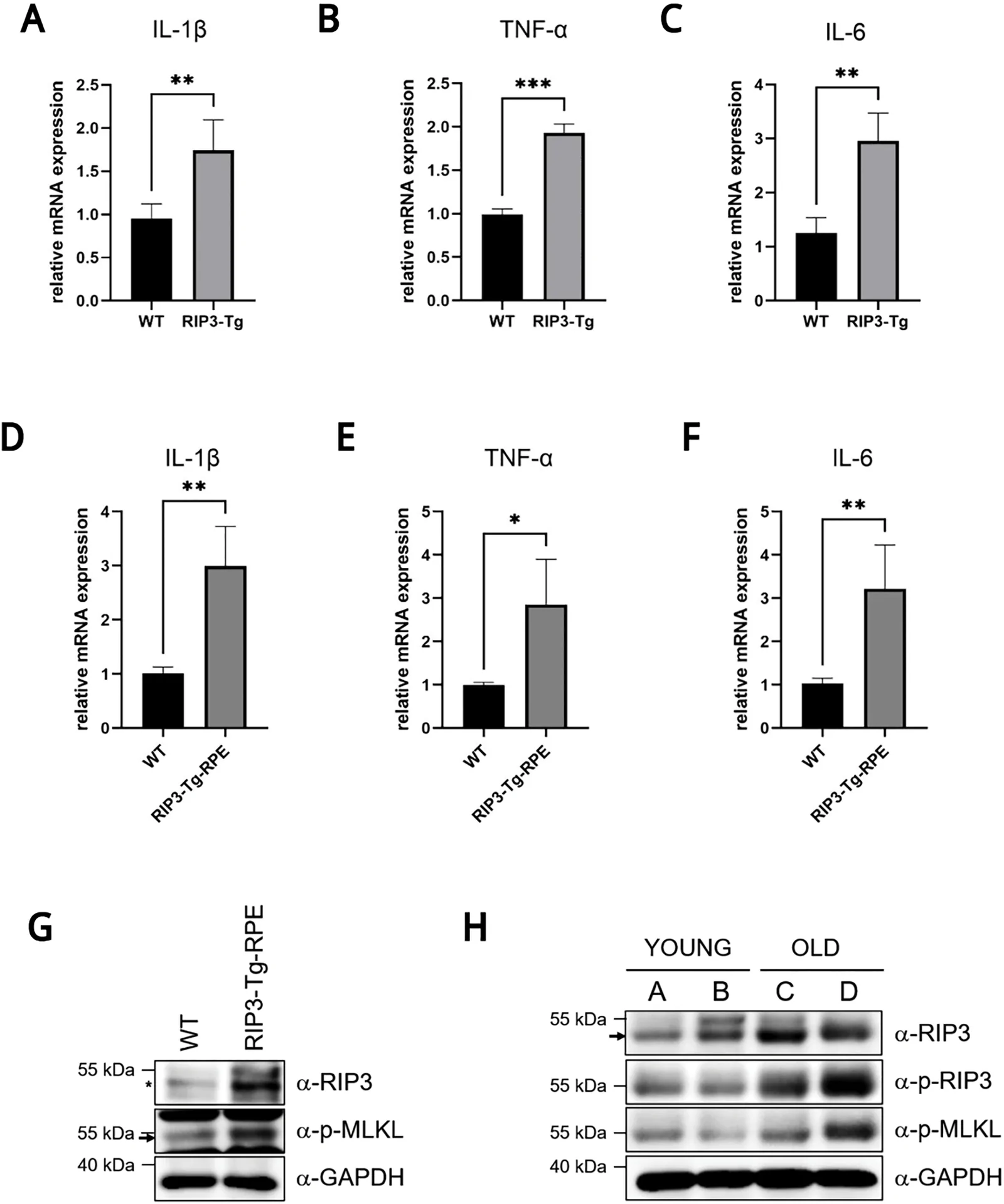
Inflammatory and RIP3–MLKL pathway signatures in RIP3-associated retinal degeneration. **(A-C)** RT-qPCR analysis of inflammatory cytokines mRNA expression from 13-month-old eyes of WT and RIP3-Tg mice (n = 3 or 4/group). **(A)** IL-1β mRNA expression **(B)** TNF-α mRNA expression **(C)** IL-6 mRNA expression. Data shown were evaluated by unpaired t-tests. Asterisks represent statistical analyses compared to the WT controls. *P < 0.05, **P < 0.01, ***P < 0.001. Data for RT-qPCR provided in S11 Table. (**D-F)** RT-qPCR analysis of inflammatory cytokines mRNA expression from 2-month-old eyes of WT and RIP3-Tg-RPE mice (n = 3 or 4/group). **(D)** IL-1β mRNA expression **(E)** TNF-α mRNA expression **(F)** IL-6 mRNA expression. Data shown were evaluated by unpaired t-tests. Asterisks represent statistical analyses compared to WT controls. *P < 0.05, **P < 0.01, ***P < 0.001. Data for RT-qPCR provided in S12 Table. (**G)** Western blot (WB) analysis for RIP3 and p-MLKL expression in 2-month-old WT and RIP3-Tg-RPE mice eyes (n = 3 per group). Asterisk represents the band indicating RIP3. Arrow indicates the band representing p-MLKL. **(H)** Western blot (WB) analysis for RIP3, p-RIP3 and p-MLKL expression in retinal tissues from four human donors. Detailed information regarding the human donors is provided in Table 2. Arrow indicates the band representing RIP3. IL-1β: Interleukin-1 Beta, TNF-α: Tumor necrosis factor-alpha, IL-6: Interleukin-6.

To investigate the molecular mechanisms underlying progressive retinal degeneration, we assessed inflammatory cytokine expression, a hallmark of dry AMD-like pathology. RT-qPCR analysis of whole eye extracts showed significant upregulation of major pro-inflammatory cytokines in RIP3-Tg mice compared to WT littermates. Specifically, mRNA levels of *IL-1β*, *TNF-α*, and *IL-6* were increased by 1.74-fold, 1.93- fold, and 2.95-fold, respectively (Fig 5A-C, S11 Table). Given these findings, we next examined whether RPE-specific RIP3 overexpression is sufficient to trigger a similar pro-inflammatory environment. In 2-month-old RIP3-Tg-RPE mice, RT-qPCR analysis revealed a marked increase in cytokine expression, with IL-1β, TNF-α, and IL-6 levels upregulated by 2.99-fold, 2.85-fold, and 3.21-fold, respectively (Fig 5D-F, S12 Table). These results confirm that RPE-specific RIP3 elevation recapitulates the pro-inflammatory cytokine profile observed in the global RIP3-Tg model. Consistent with these inflammatory changes, western blot analysis of retinal tissues from 2-month-old mice confirmed that RIP3-Tg-RPE mice displayed significantly higher levels of both RIP3 and the necroptosis marker, p-MLKL (Fig 5G). Collectively, these data indicate that elevated RIP3 expression specifically within the RPE contributes to a pro-inflammatory and necroptotic environment, supporting a functional link between RIP3 mediated RPE stress and the progression of retinal degeneration.

Finally, to further evaluate the translational and clinical relevance of our transgenic mouse models, we investigated whether the necroptotic pathway is activated in the human retina during aging. We analyzed the expression of key necroptosis-associated proteins (RIP3, p-RIP3, and p-MLKL) in human retinal/RPE donor tissues. While basal expression of these necroptosis markers was generally low in young donors (20s), it was markedly elevated in aged donors (80s) (Fig 5H). Collectively, these findings from human retina/RPE tissues are consistent with our observations in the transgenic mouse models and support the biological and translational relevance of RIP3-MLKL pathway activation in age-associated retinal degeneration.

## Discussion

In this study, we established complementary RIP3 transgenic mouse models that exhibit gradually progressive and accelerated forms of RIP3-associated retinal degeneration. The major contribution of this work is the development of an *in vivo* model system in which sustained increases in RIP3 expression are associated with progressive retinal degeneration, inflammatory activation, and necroptotic cell death. RIP3-Tg mice, which exhibit basal ectopic RIP3 expression, developed a slowly progressive retinal phenotype over time. In contrast, RIP3-Tg-RPE mice, which carry this basal expression together with additional RPE-enriched RIP3 expression driven by Best1-Cre, showed markedly accelerated retinal abnormalities at an early age. Together, these findings suggest that increased RIP3 expression burden is associated with both the onset and progression rate of retinal degeneration.

Necroptosis has emerged as a regulated form of inflammatory cell death implicated in multiple degenerative diseases, including retinal disorders [17,18]. Previous studies have reported increased RIP3 expression in degenerating RPE and demonstrated that inhibition of RIP3 attenuates RPE loss [19–21]. However, whether sustained RIP3 upregulation contributes to the development of progressive retinal pathology *in vivo* has remained unclear. Our findings extend these previous observations by demonstrating that ectopic RIP3 expression is associated with progressive retinal degeneration accompanied by pro-inflammatory cytokine induction and RIP3-MLKL pathway activation. In RIP3-Tg mice, retinal degeneration was accompanied by increased expression of IL-1β, TNF-α, and IL-6 [22–24], as well as tissue-level TUNEL and p-MLKL signal changes at later disease stages. In RIP3-Tg-RPE mice, early retinal abnormalities were accompanied by increased pro-inflammatory cytokine expression and elevated RIP3 and p-MLKL protein levels. These results support a close association between increased RIP3 expression, inflammatory activation, and necroptosis-related signaling during retinal degeneration.

The two RIP3 transgenic lines showed distinct patterns of disease progression. RIP3-Tg mice developed gradual retinal degeneration over time [25], whereas RIP3-Tg-RPE mice showed early and accelerated retinal pathology accompanied by an additional increase in RIP3 expression in the RPE compartment. These findings suggest that the overall burden and tissue enrichment of RIP3 expression are closely associated with the timing and severity of retinal degeneration. Because Cre-independent recombination was observed in the RIP3-Tg line, the difference between the two models should be interpreted as a quantitative and spatial difference in RIP3 expression rather than as a strict comparison between systemic and purely RPE-specific expression. Together, these models provide a useful system for evaluating how RIP3 expression burden and RPE enrichment influence the progression kinetics of retinal degeneration in vivo.

Compared with conventional chemically induced models, such as sodium iodate injury or light-induced retinal damage [26–31], these RIP3 transgenic models offer a complementary approach for studying retinal degeneration. Acute injury models are useful for inducing rapid and reproducible retinal damage, but they do not fully capture the chronic and progressive nature of human dry AMD or the interplay between cell death and inflammation. In contrast, the RIP3-Tg model provides a system for examining gradually progressive retinal pathology associated with sustained RIP3 expression, while the RIP3-Tg-RPE model enables investigation of accelerated retinal degeneration associated with additional RPE-enriched RIP3 expression. These models should not be considered complete phenotypic replicas of human AMD, as they do not reproduce all defining pathological hallmarks of the disease, such as typical drusen formation. Rather, they represent mechanism-focused models of RIP3-associated retinal degeneration with selected AMD-relevant features, including retinal thinning, RPE-associated alterations, inflammatory activation, and necroptosis-related signaling.

The relevance of RIP3–MLKL pathway activation to human retinal degeneration is further supported by our analysis of human donor RPE tissue samples. In these samples, RIP3, phosphorylated RIP3, and phosphorylated MLKL were detectable and showed a tendency toward higher expression in aged donor tissues compared with young donor tissues, although donor-to-donor variability was observed. These findings provide supportive evidence that RIP3–MLKL pathway activation may be relevant to aged and degenerative human retinal tissue, although further validation in larger human cohorts will be required.

Several limitations should be considered when interpreting these findings. First, OFMT was used to provide indirect functional evidence of visual impairment rather than a definitive physiological measure, and performance in this assay may be influenced by non-visual factors, including anxiety-like behavior and locomotor activity. Although increased fecal pellets counts suggest that anxiety-like behavior may have contributed to altered open-field behavior, future studies using electroretinography or optokinetic response testing will be required for more objective functional characterization. Second, although TUNEL staining, p-MLKL immunostaining, and Western blot analysis support cell death and RIP3–MLKL pathway activation, these assays do not establish definitive causality or identify the specific cell types responsible for pathway activation. Pharmacological or genetic inhibition of RIP3/MLKL will be needed to determine whether retinal degeneration in these models is functionally dependent on necroptosis-related signaling. Finally, the RPE-related findings should be interpreted as RPE-associated alterations rather than definitive evidence of uniform RPE cell loss, because patchy RPE65 staining and qualitative RPE flatmount abnormalities do not provide unbiased whole-mount quantification of RPE cell density.

From a therapeutic perspective, our findings support necroptosis-related signaling as a potential target for slowing retinal degeneration. Although dry AMD and related retinal degenerative diseases involve complex and multifactorial mechanisms, the present models provide a useful in vivo system for examining the contribution of RIP3-associated signaling to retinal pathology. Overall, this study establishes complementary RIP3 transgenic mouse models that capture gradually progressive and accelerated forms of retinal degeneration and may offer valuable platforms for investigating both the mechanisms and progression kinetics of dry AMD-like pathology and may facilitate the development of therapeutic strategies targeting RIP3–MLKL pathway activation.

## Supporting information

S1 Fig Representative OCT image showing quantitative thickness measurements of total retina, ONL, and PRL. (DOCX)

S2 Fig Representative diagram showing the set-up of equipment for open-field mirror test. (DOCX)

S3 Fig Increased number of fecal pellets in 13-month-old RIP3-Tg mice. (DOCX)

S4 Fig RIP3-mediated RPE stress and RPE65 downregulation in RIP3-Tg mice. (DOCX)

S5 Fig Increased number of fecal pellets in 3-month-old RIP3-Tg-RPE mice. (DOCX)

S1 Table Extended RT-qPCR data for Fig. 1D. (XLSX)

S2 Table Extended Total, ONL, PRL thickness data for Fig. 2C-E. (XLSX)

S3 Table Extended OFMT data for Fig. 2G-I. (XLSX)

S4 Table Extended fecal pellet data for S3 Fig. (XLSX)

S5 Table Extended Total thickness data for Fig. 4C. (DOCX)

S6 Table Extended ONL thickness data for Fig. 4D. (XLSX)

S7 Table Extended PRL thickness data for Fig. 4E. (XLSX)

S8 Table Extended number of ONL cell count data for Fig. 4G. (XLSX)

S9 Table Extended OFMT data for Fig 4J and K. (XLSX)

S10 Table Extended fecal pellet data for S5 Fig. (XLSX)

S11 Table Extended RT-qPCR data for Fig 5A-C. (XLSX)

S12 Table Extended RT-qPCR data for Fig 5D-F. (XLSX)

S1 Raw images Uncropped original scans for RT-PCR and Western blots. (PDF)
