# Supplementary material for: Generation of dual mouse models of retinal degeneration with slow and rapid progression driven by ectopic RIP3 expression
Source: PLoS One. 2026 Aug 20;21(8):e0356284. doi: 10.1371/journal.pone.0356284 (PMC13492740; doi:10.1371/journal.pone.0356284)

Uncropped Scans of RT-PCR Blots in Fig 1C

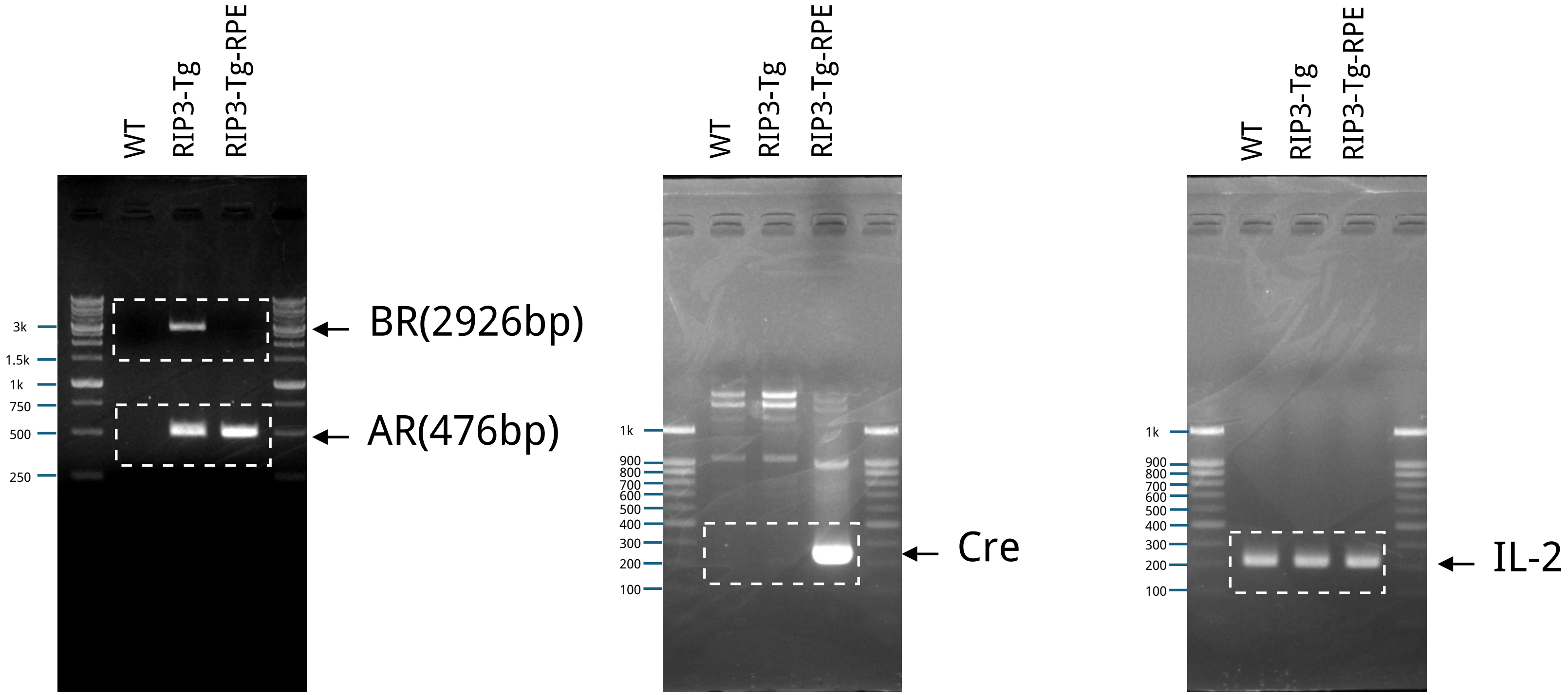

Uncropped Scans of Western Blots in Fig 1E

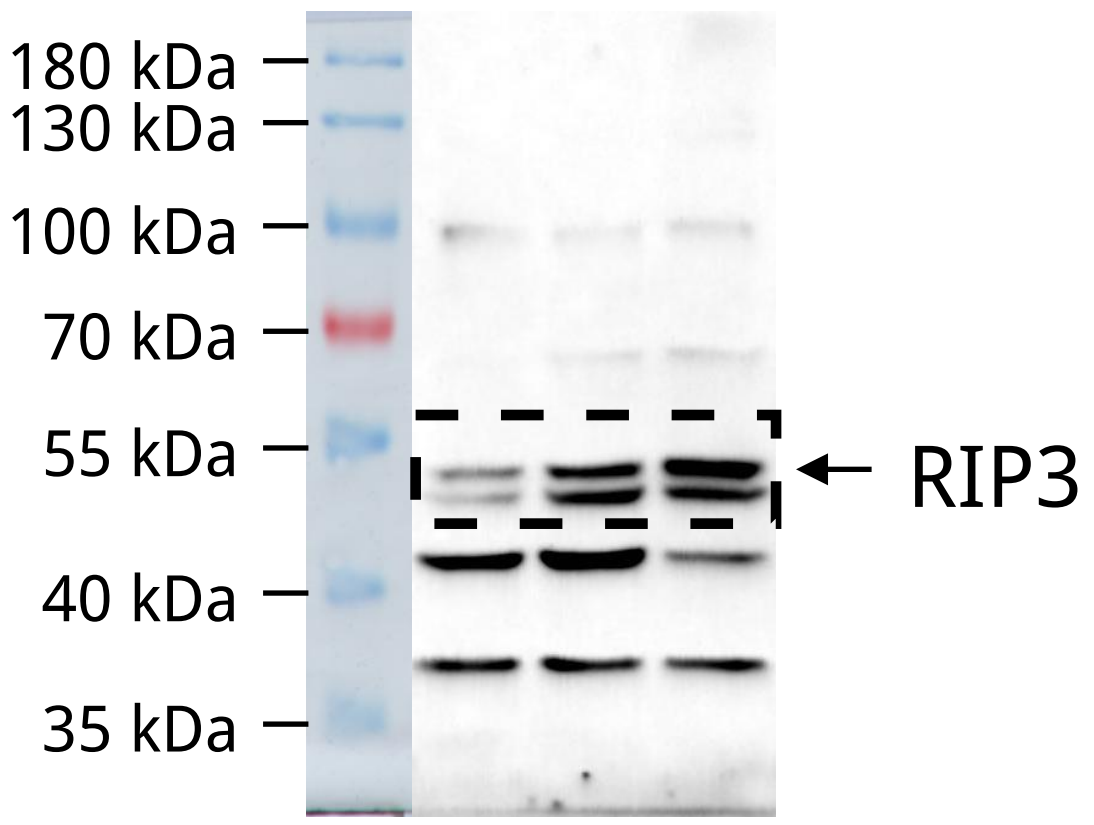

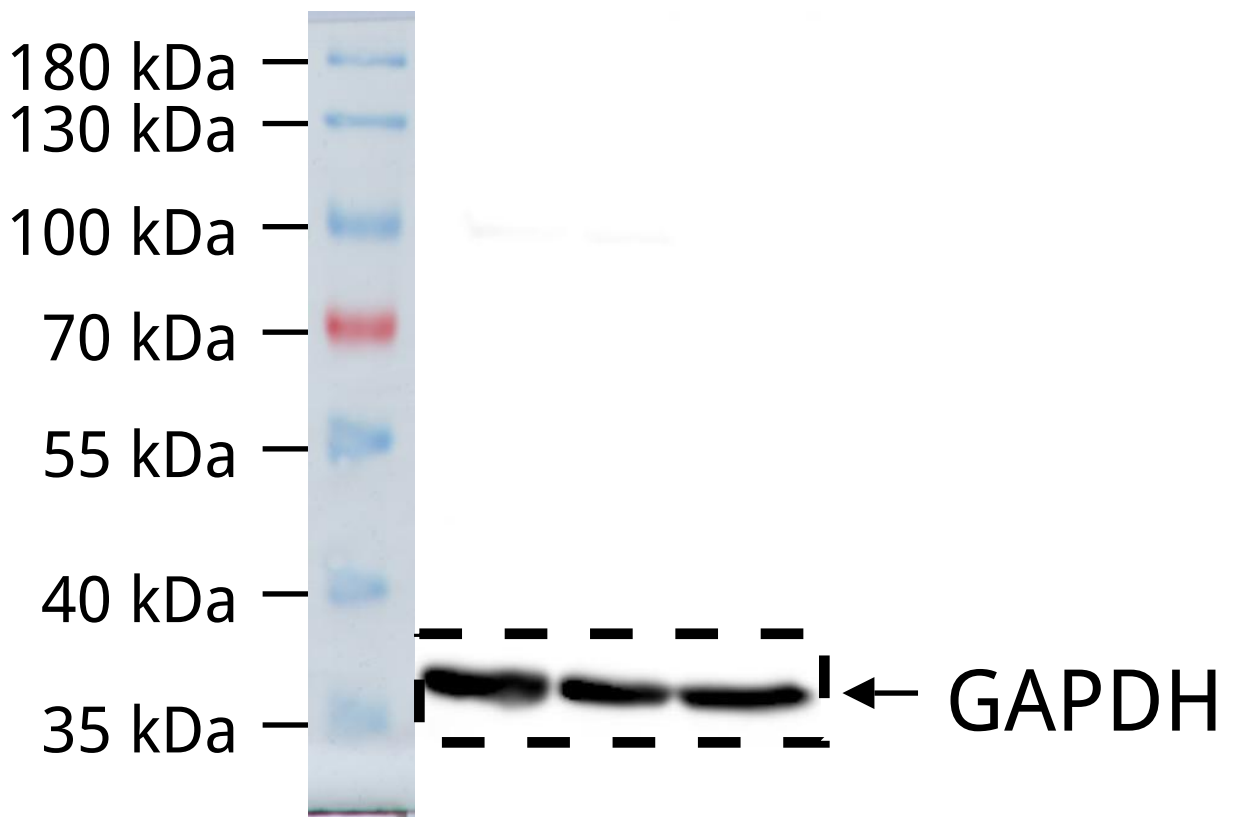

Uncropped Scans of Western Blots in S4 Fig

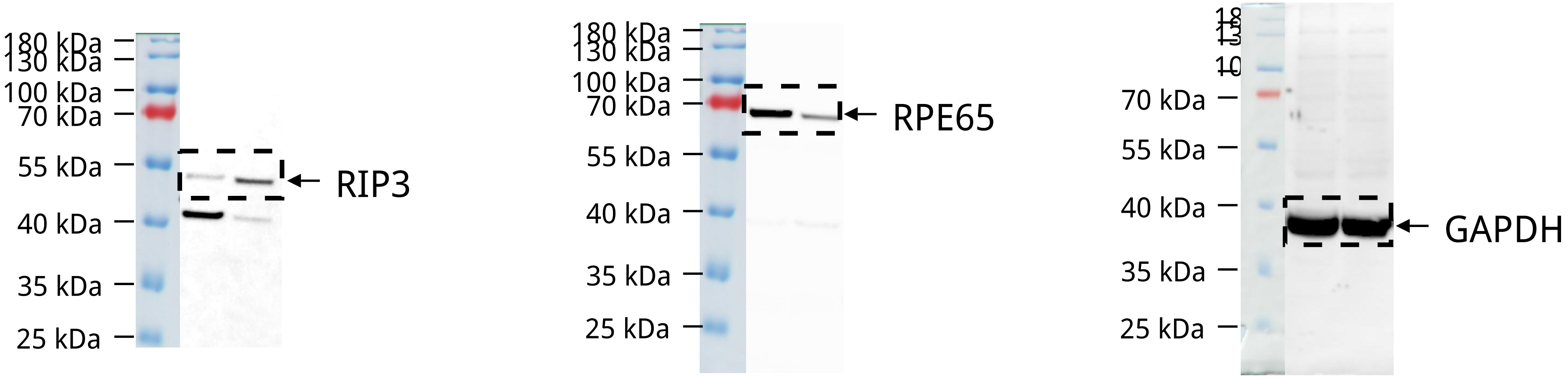

Uncropped Scans of Western Blots in Fig 5G

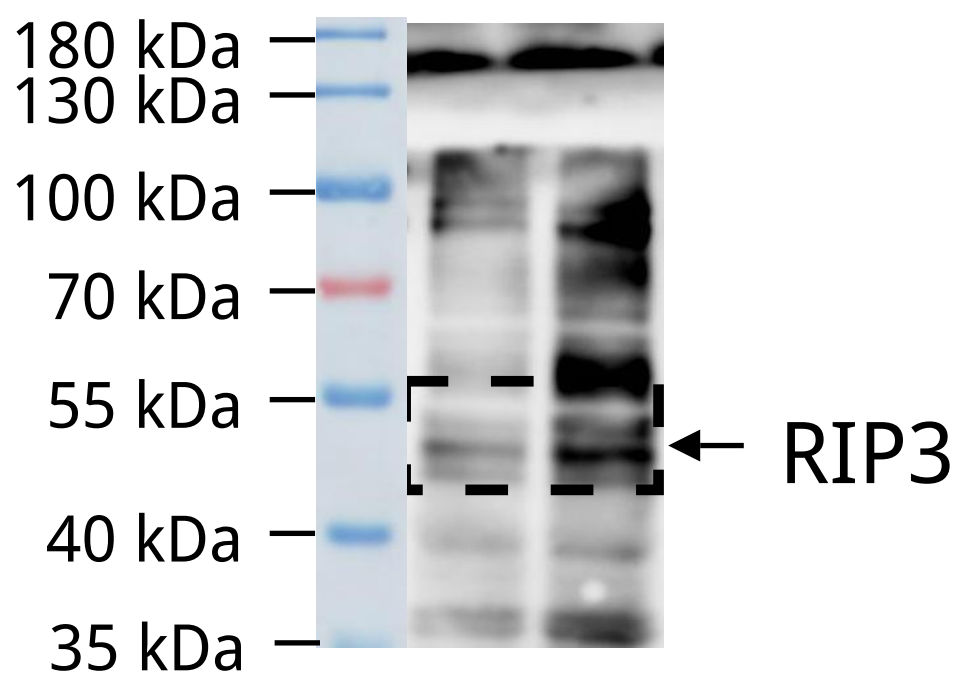

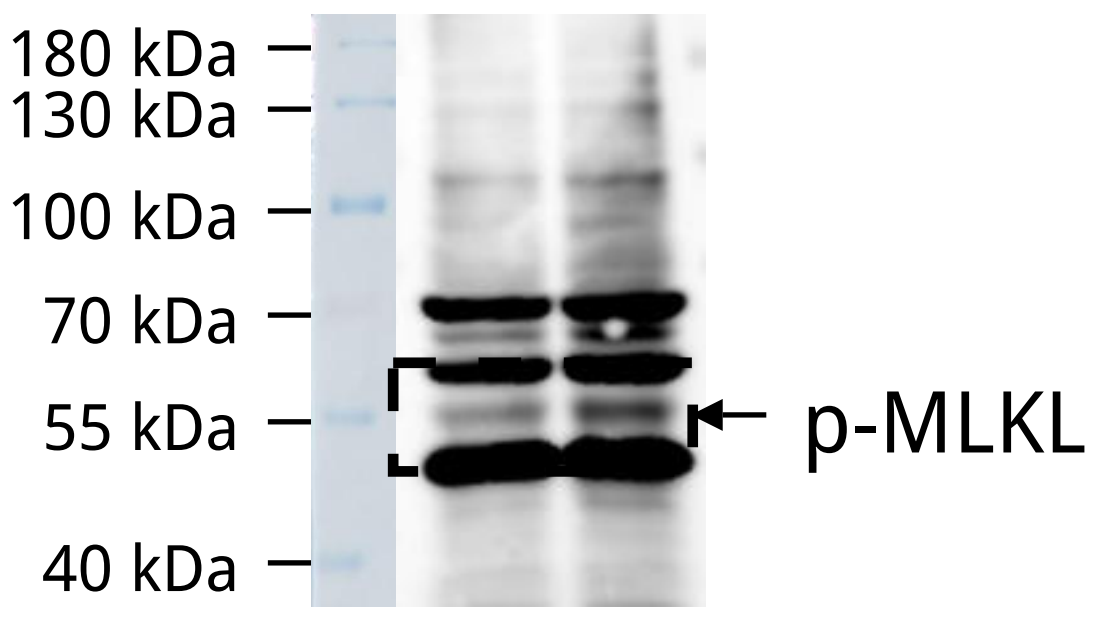

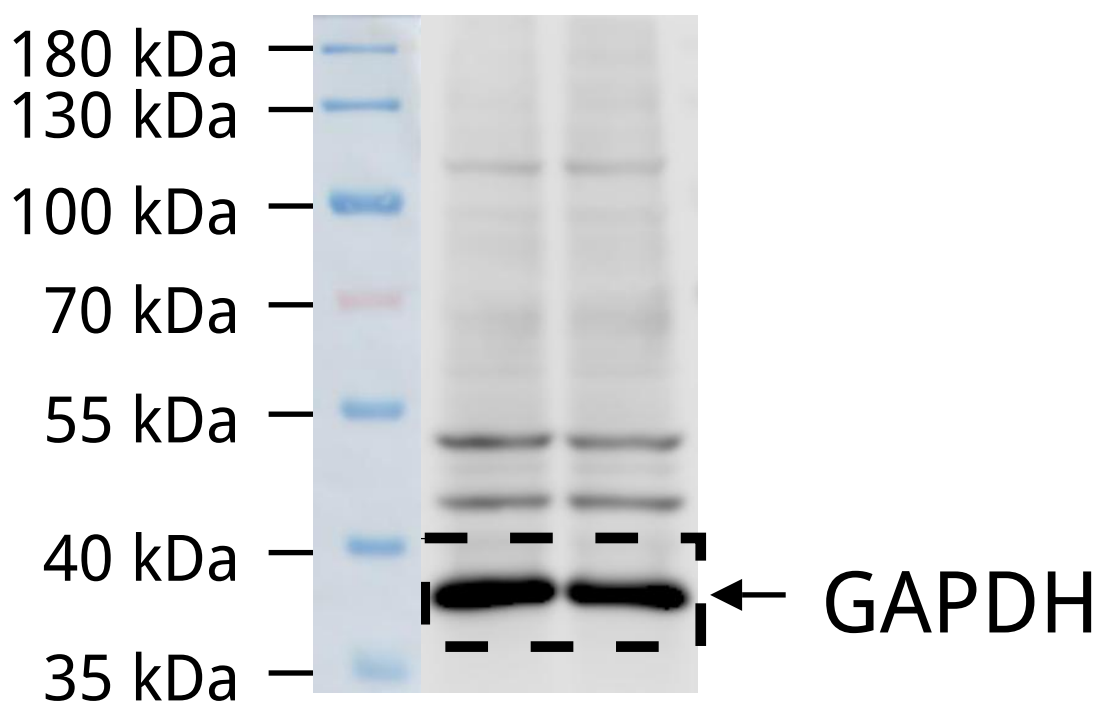

Uncropped Scans of Western Blots in Fig 5H

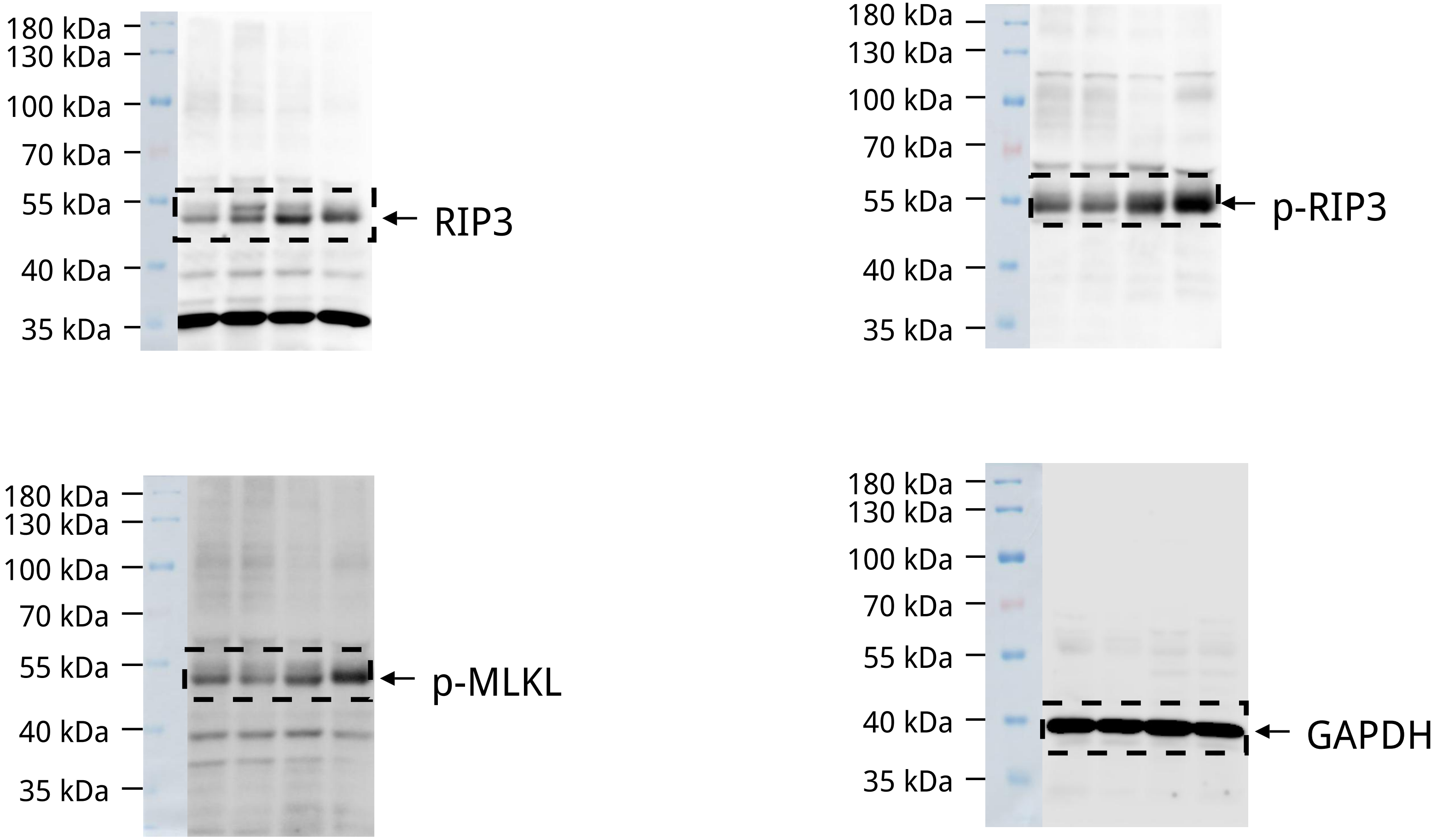

Supplement: Supplementary file 1 [file pone.0356284.s018.pdf]
