# Supplementary material for: Generation of dual mouse models of retinal degeneration with slow and rapid progression driven by ectopic RIP3 expression
Source: PLoS One. 2026 Aug 20;21(8):e0356284. doi: 10.1371/journal.pone.0356284 (PMC13492740; doi:10.1371/journal.pone.0356284)
Supplement: Supplementary file 2 [file pone.0356284.s001.docx]

**S1 Fig.** **Representative OCT image showing quantitative thickness measurements of total retina, ONL, and PRL.** *Abbreviation: ONL: outer nuclear layer, PRL: photoreceptor layer.*
