# Supplementary material for: Generation of dual mouse models of retinal degeneration with slow and rapid progression driven by ectopic RIP3 expression
Source: PLoS One. 2026 Aug 20;21(8):e0356284. doi: 10.1371/journal.pone.0356284 (PMC13492740; doi:10.1371/journal.pone.0356284)
Supplement: Supplementary file 3 [file pone.0356284.s002.docx]

**S2 Fig.** **Representative diagram showing the set-up of equipment for open-field mirror test.** The open-field mirror test with 9 subzones. Z2: mirrored area, Z8: reversed mirror, Z4 and Z6: null zones.
