# Supplementary material for: Generation of dual mouse models of retinal degeneration with slow and rapid progression driven by ectopic RIP3 expression
Source: PLoS One. 2026 Aug 20;21(8):e0356284. doi: 10.1371/journal.pone.0356284 (PMC13492740; doi:10.1371/journal.pone.0356284)
Supplement: Supplementary file 5 [file pone.0356284.s004.docx]

**S4 Fig.** **RIP3-mediated RPE stress and RPE65 downregulation in RIP3-Tg mice.** Western blot (WB) analysis for RIP3 and RPE65 expression in 5-month-old WT and RIP3-Tg mice eyes (n=3 per group). Arrow indicates the band representing RIP3.
