# Supplementary material for: Generation of dual mouse models of retinal degeneration with slow and rapid progression driven by ectopic RIP3 expression
Source: PLoS One. 2026 Aug 20;21(8):e0356284. doi: 10.1371/journal.pone.0356284 (PMC13492740; doi:10.1371/journal.pone.0356284)
Supplement: Supplementary file 7 [file pone.0356284.s010.docx]

**S5 Fig.** **Increased number of fecal pellets in 3-month-old RIP3-Tg-RPE mice.** Number of fecal pellets were counted during the open-field mirror test (n=6/each group). Data shown were evaluated by two tailed unpaired t-test. Asterisks represent statistical analyses compared to WT controls. *P<0.05, **P<0.01. Data measurements listed in S10 Table.

Fecal pellet No.
